# SMTP-44D Attenuates Renal Inflammatory/Oxidative Dysfunction in a Mouse Model of Ischemia–Reperfusion-Induced Acute Kidney Injury

**DOI:** 10.3390/ijms27198506

**Published:** 2026-09-24

**Authors:** Keita Shibata, Keiji Hasumi, Koji Nobe

**Affiliations:** 1 Department of Pharmacology, Graduate School of Pharmacy, Showa University, Tokyo 142-8555, Japan; kojinobe@pharm.showa-u.ac.jp; 2 Pharmacological Research Center, Showa Medical University, 1-5-8 Hatanodai, Shinagawa-ku, Tokyo 142-8555, Japan; 3 Department of Applied Biological Science, Tokyo University of Agriculture and Technology, 3-5-8 Saiwaicho, Fuchu, Tokyo 183-8509, Japan; hasumi@tms-japan.co.jp; 4 Division of Research and Development, TMS Co., Ltd., Fuchu, Tokyo 183-0055, Japan

**Keywords:** acute kidney injury, SMTP-44D, anti-inflammatory activity, antioxidant activity

## Abstract

Acute kidney injury (AKI) is a critical clinical challenge for which effective pharmacological therapies remain limited. Inflammation and oxidative stress play central roles in its pathogenesis. Here, we investigated the effects of *Stachybotrys microspora* triprenyl phenol-44D (SMTP-44D), a soluble epoxide hydrolase inhibitor that, unlike its analog SMTP-7, lacks thrombolytic activity, in a mouse model of renal ischemia–reperfusion (I/R)-induced AKI. SMTP-44D was administered either during or prior to ischemia. SMTP-44D improved renal functional parameters, including blood urea nitrogen, serum creatinine, and fractional sodium excretion in both protocols, with a protocol-dependent difference in creatinine clearance. An attenuation of tubular morphological alterations was observed when SMTP-44D was administered during ischemia, whereas pre-ischemic administration failed to demonstrate apparent morphological improvement. Mechanistic analysis was conducted using the pre-ischemic administration protocol. Urinary neutrophil gelatinase-associated lipocalin and kidney injury molecule 1 were significantly reduced. SMTP-44D also suppressed renal tumor necrosis factor alpha, interleukin-1 beta, and NADPH oxidase-1 levels. Collectively, these functional and molecular findings suggest that SMTP-44D provides anti-inflammatory and antioxidant renoprotection, while evidence supporting structural protection is lacking in the pre-ischemic administration protocol. SMTP-44D warrants further investigation as a potential therapeutic strategy for I/R-induced AKI.

## 1. Introduction

Acute kidney injury (AKI), characterized by an abrupt decline in kidney function and the retention of uremic toxins, poses a global public health challenge and is associated with substantial mortality rates [1,2,3,4]. Clinically, AKI is diagnosed primarily on the basis of changes in serum creatinine and urine output [5]. AKI arises from diverse etiologies, ranging from sepsis to nephrotoxin exposure. Among these, ischemia–reperfusion injury (I/R) represents a predominant cause and is frequently observed in clinical settings such as renal transplantation, partial nephrectomy, and cardiac surgery [2,6,7,8,9,10]. According to recent estimates, I/R contributes to approximately 20–30% of all AKI cases among patients who are hospitalized [11]. The pathophysiology of I/R-induced AKI involves two critical phases. The ischemic phase leads to metabolic dysfunction and ATP depletion in renal tubular epithelial cells [12,13]. This is followed by the reperfusion phase, during which the sudden restoration of oxygen supply induces a surge in reactive oxygen species (ROS), resulting in cellular organelle damage and activating inflammatory cascades [14,15]. These processes trigger renal tubular cell injury and death, accompanied by peritubular endothelial dysfunction and inflammatory cell infiltration [7,16,17,18]. Various pharmacological approaches, including antioxidants, anti-inflammatory agents, vasodilators, and anti-apoptotic therapies, have been investigated as potential treatments for AKI. Although many have demonstrated renoprotective effects in experimental models, none has yet been established as an effective pharmacological therapy for I/R-induced AKI. Despite improvements in perioperative management, effective pharmacological interventions targeting the core mechanisms of I/R-induced injury remain lacking [19,20,21,22]. Accordingly, prevention of excessive inflammation and cell death represents a principal therapeutic strategy [23], underscoring the urgent need to identify novel therapeutic agents.

*Stachybotrys microspora* triprenyl phenols (SMTPs) are a group of small-molecule metabolites produced by the fungus *S. microspore* [24]. These compounds are notable for their ability to modulate multiple biological pathways. In a previous study, among the SMTPs, SMTP-7 has demonstrated therapeutic potential in various disease models, including embolic stroke [25,26] and AKI [27]. The efficacy of SMTP-7 is attributed to its anti-inflammatory and antioxidant mechanisms, which are partly mediated by the inhibition of soluble epoxide hydrolase (sEH). This inhibition increases levels of epoxyeicosatrienoic acids (EETs), endogenous lipid mediators known to possess potent cytoprotective properties [28]. EETs have been reported to suppress inflammatory signaling, attenuate oxidative stress, improve endothelial function, and preserve renal microvascular perfusion, thereby contributing to protection against I/R-induced tissue injury. These biological effects provide a mechanistic rationale for targeting the sEH/EET pathway in AKI. However, SMTP-7 also exhibits strong thrombolytic activity by enhancing plasminogen activation [26] (Figure 1). Although thrombolytic activity is beneficial in thrombotic conditions such as cerebral infarction, it may be unnecessary for the treatment of AKI, in which thrombosis is not the primary pathology, and could potentially increase bleeding risk in perioperative settings. Several SMTP compounds inhibit sEH [29,30], and this inhibition may account for their anti-inflammatory activity [30]. The thrombolytic activity of SMTP-7 is independent of sEH inhibition [31] and is mediated through modulation of plasminogen conformation, which facilitates plasminogen-fibrin binding and plasminogen activation by plasminogen activators [29]. In contrast, SMTP-44D has very low potency in modulating plasminogen conformation [30] and is therefore essentially inactive in promoting thrombolysis [25]. These findings suggest that the structural modifications introduced into SMTP-44D selectively reduced its interaction with plasminogen while preserving its sEH inhibitory activity. This pharmacological profile may represent a clinically advantageous property for the treatment of AKI, where thrombolytic activity is unnecessary and may increase the risk of bleeding.

In this study, we focused on SMTP-44D, a distinct member of the SMTP family. SMTP-44D has demonstrated therapeutic potential in various disease models, including diabetic neuropathy [32,33,34], diabetic retinopathy [35], and diabetic nephropathy [36], primarily owing to its anti-inflammatory and antioxidative properties. In contrast to SMTP-7, SMTP-44D does not exhibit thrombolytic activity but retains potent sEH inhibitory activity (Figure 1). This pharmacological profile enables specific evaluation of the therapeutic potential of sEH inhibition in AKI without the confounding effects of thrombolysis. Furthermore, the molecular weight of SMTP-44D is lower than that of SMTP-7 [25]. However, whether this difference translates into pharmacokinetic or tissue-distribution advantages has not been determined. Given its mechanism of action, SMTP-44D may represent a potential therapeutic candidate for AKI by targeting key pathological processes involved in the disease. However, the therapeutic efficacy of SMTP-44D in the context of AKI has not been fully established. Moreover, whether its anti-inflammatory and antioxidant properties directly contribute to renal protection in this setting remains unknown. Consequently, this study aimed to evaluate the renoprotective effects of SMTP-44D in AKI and to elucidate the underlying mechanisms, with a specific focus on inflammation and oxidative stress.

## 2. Results

### 2.1. Renal Function Based on Urinary Parameters and Kidney Weight

I/R injury consistently induced renal damage in both experimental protocols. In the experiment involving treatment during ischemia, the control group exhibited substantial elevations in urinary albumin (Ualb) (sham: 0.3 ± 0.1 vs. control: 1.3 ± 0.2 mg/h/kg), urine flow (UF) (sham: 35.7 ± 4.8 vs. control: 88.9 ± 11.9 μL/min/kg), and kidney weight (KW) (sham: 9.0 ± 0.4 vs. control: 13.9 ± 0.6 mg/g) (Figure 2). Administration of SMTP-44D (30 mg/kg) during ischemia did not significantly alter the levels of Ualb (1.3 ± 0.3 mg/h/kg), UF (103.2 ± 33.7 μL/min/kg), and KW (12.3 ± 1.2 mg/g) compared to those in the control group. In the experiment involving treatment prior to ischemia, significant increases in Ualb (sham: 0.2 ± 0.1 vs. control: 0.8 ± 0.1 mg/h/kg) and KW (sham: 8.9 ± 0.5 vs. control: 13.5 ± 1.3 mg/g) but not UF (sham: 47.1 ± 6.8 vs. control: 65.4 ± 7.3 μL/min/kg) were detected. Pre-treatment with SMTP-44D (30 mg/kg) significantly attenuated the increase in Ualb to 0.5 ± 0.1 mg/h/kg, whereas KW remained unchanged (13.2 ± 0.9 mg/g). Moreover, SMTP-44D administration resulted in a notable increase in UF (109.0 ± 19.4 μL/min/kg) compared with that in the control group. Given the protective effect on Ualb observed at 30 mg/kg, a lower dose of SMTP-44D (3 mg/kg) was further evaluated to explore whether the effect was retained at a lower dose. Treatment with 3 mg/kg resulted in a partial reduction in Ualb (0.6 ± 0.1 mg/h/kg), although no statistically significant differences were observed between the 3 mg/kg and 30 mg/kg groups. The 30 mg/kg group showed numerically greater improvement than the 3 mg/kg group. These findings do not demonstrate dose dependence, and a definitive dose–response relationship could not be established.

### 2.2. Renal Function Based on Serum Parameters

Serum biochemical analyses indicated that renal filtration function was severely impaired by I/R injury in both experimental protocols. In the experiment involving treatment during ischemia, the control group showed markedly elevated levels of blood urea nitrogen (BUN) (sham: 38.4 ± 2.7 vs. control: 192.2 ± 44.3 mg/dL), creatinine concentrations in serum (Scr) (sham: 0.3 ± 0.0 vs. control: 1.7 ± 0.4 mg/dL), and fractional excretion of sodium (FE_Na_) (sham: 0.9 ± 0.0 vs. control: 5.2 ± 2.1%), along with a substantial decline in creatinine clearance (Ccr) (sham: 2.8 ± 0.2 vs. control: 1.2 ± 0.4 mL/min/kg) (Figure 3). Treatment with SMTP-44D (30 mg/kg) during ischemia markedly attenuated these alterations, with BUN, Scr, and FE_Na_ levels reduced to 55.7 ± 14.2 mg/dL, 0.6 ± 0.1 mg/dL, and 1.2 ± 0.1%, respectively, and with Ccr elevated to 2.3 ± 0.4 mL/min/kg. In the experiment involving treatment prior to ischemia, renal dysfunction comparable to the intra-ischemic treatment was observed, as evidenced by increased BUN (sham: 49.2 ± 2.9 vs. control: 126.5 ± 26.8 mg/dL), Scr (sham: 0.2 ± 0.0 vs. control: 0.7 ± 0.1 mg/dL), FE_Na_ (sham: 0.6 ± 0.1 vs. control: 2.5 ± 0.4%), together with decreased Ccr (sham: 9.7 ± 3.8 vs. control: 1.0 ± 0.2 mL/min/kg). Pre-treatment with SMTP-44D (30 mg/kg) significantly lowered BUN (43.3 ± 6.9 mg/dL), Scr (0.4 ± 0.1 mg/dL), and FE_Na_ (0.6 ± 0.2%). Regarding Ccr, although the increase (5.1 ± 1.5 mL/min/kg) did not reach statistical significance compared with that in the control group, the levels were restored to a range comparable to the sham group. Administration of the lower dose (3 mg/kg) produced improvements in BUN (52.6 ± 6.1 mg/dL), Scr (0.4 ± 0.0 mg/dL), and FE_Na_ (0.8 ± 0.1%) but not Ccr (3.4 ± 0.5 mL/min/kg).

### 2.3. Morphological Assessment of Tubular Injury

The morphology of the outer stripe of the outer medulla, where ischemia–reperfusion injury developed prominently, was assessed to evaluate the impact of SMTP-44D on renal tubular injury. Representative H.E.-stained images are shown in Figure 4. In the treatment during ischemia protocol (Figure 4A), the control group exhibited marked changes in renal tubule morphology, including tubular dilation and tubular cast formation, compared to the sham-operated group. Treatment with SMTP-44D attenuated these morphological changes in the renal tubules. In the treatment prior to ischemia protocol (Figure 4B), tubular injury development was milder than that observed in the treatment during ischemia protocol. Under these experimental conditions, an apparent treatment-related improvement in tubular morphology was not observed with SMTP-44D. Therefore, the histopathological findings from this protocol do not provide evidence of structural protection by SMTP-44D. Possible factors contributing to the difference in the severity of histological injury between the two experimental protocols are discussed Section 3.

### 2.4. Characterization of the Effects of Pre-Ischemic Treatment with SMTP-44D

To further investigate the protective mechanism of SMTP-44D, urinary tubular injury markers were evaluated using samples from the treatment prior to the ischemia protocol, in which improvements in renal function, including albuminuria, were observed. Following I/R injury, urinary excretion levels of neutrophil gelatinase-associated lipocalin (NGAL) and kidney injury molecule 1 (KIM-1) were markedly increased in the control group (NGAL; 24.8 ± 4.2 ng/μg Cr, KIM-1; 199.2 ± 41.8 pg/μg Cr) compared with those in the sham group (NGAL; 2.0 ± 0.4 ng/μg Cr, KIM-1; 0.3 ± 0.1 pg/μg Cr) (Figure 5). Pre-treatment with SMTP-44D (30 mg/kg) significantly attenuated the levels of NGAL and KIM-1 to 11.1 ± 1.1 ng/μg Cr and 41.5 ± 8.3 pg/μg Cr, respectively.

### 2.5. Anti-Inflammatory and Antioxidant Effects of SMTP-44D

The levels of pro-inflammatory cytokines and an oxidative stress marker in kidney tissues were examined, focusing on the treatment prior to the ischemia protocol. I/R injury induced a marked inflammatory and oxidative response, characterized by substantial increases in renal tumor necrosis factor alpha (TNF-α) (sham: 0.6 ± 0.0 vs. control: 0.9 ± 0.1 pg/mg protein), interleukin (IL)-1β (sham: 10.9 ± 0.4 vs. control: 17.1 ± 2.3 pg/mg protein), IL-6 (sham: 0.5 ± 0.0 vs. control: 2.9 ± 1.0 pg/mg protein), and NADPH oxidase-1 (NOX-1) (sham: 42.3 ± 0.9 vs. control: 59.3 ± 3.6 pg/mg protein) (Figure 6). Administration of SMTP-44D (30 mg/kg) significantly attenuated the elevations in TNF-α (0.7 ± 0.0 pg/mg protein), IL-1β (9.7 ± 0.6 pg/mg protein), and NOX-1 (39.1 ± 1.6 pg/mg protein) compared to the control group. Regarding IL-6, although the level in the SMTP-44D group was reduced to approximately 55% (1.6 ± 0.4 pg/mg protein) of that in the control group, this reduction did not reach statistical significance and should therefore be interpreted with caution.

## 3. Discussion

In this study, we investigated the effects of SMTP-44D on renal I/R injury in a mouse model of AKI using two drug administration protocols: administration prior to and during ischemia. SMTP-44D improved several renal functional parameters following I/R injury in both protocols. Specifically, SMTP-44D treatment improved Ualb, BUN, Scr, and FE_Na_, with protocol-dependent changes in Ccr. We further investigated the SMTP-44D effects in the pre-ischemia protocol, and found that SMTP-44D reduced tubular injury markers (NGAL and KIM-1). These molecular markers were included because they provide sensitive and objective information regarding acute tubular injury [37,38,39]. In addition, SMTP-44D suppressed renal TNF-α, IL-1β, and NOX-1 levels. However, unlike in the treatment during the ischemia protocol, histopathological protection was observed in the treatment during the ischemia protocol. Therefore, these findings support functional and molecular but not structural protective effects of SMTP-44D. Furthermore, we have shown that these beneficial effects are associated with suppression of inflammatory and oxidative stress signaling pathways in the kidney. Functional and molecular findings provide complementary information regarding the severity and characteristics of AKI [21]. Renal injury biomarkers may also detect tubular injury that is not fully reflected by conventional functional indices, highlighting the complementary roles of these assessments [40,41].

In addition to the functional and molecular assessments, histological preservation represents an important component of the multidimensional evaluation of renal protection. In the treatment during ischemia protocol, SMTP-44D attenuated I/R-induced morphological alterations, including tubular dilation and tubular cast formation. These observations were accompanied by improvements in BUN, Scr, Ccr, and FE_Na_. In contrast, the treatment prior to ischemia protocol produced only mild histological injury, and an apparent treatment-related morphological improvement was not observed with SMTP-44D. Accordingly, the findings from this protocol should primarily be interpreted as evidence of functional and molecular protection rather than structural protection.

The apparent difference in the severity of histological injury between the two independently conducted protocols may, at least in part, have been influenced by differences in the volume and rate of intravenous fluid administration. A total of 1 mL/kg was infused for 30 min in the treatment during ischemia protocol, whereas a total of 4 mL/kg was injected for 2 min in the treatment prior to ischemia protocol. Previous studies have demonstrated that fluid administration can influence the severity of renal I/R injury [42,43]; therefore, the larger and more rapid fluid administration in the latter protocol may have contributed to the relatively mild structural injury. Importantly, however, within the treatment prior to ischemia protocol, the vehicle-treated I/R group and the SMTP-44D-treated groups received identical infusion volumes and administration schedules. Thus, fluid administration may have influenced the overall severity of structural injury in this protocol but is unlikely to explain the differences between the vehicle-treated and SMTP-44D-treated groups in renal functional parameters and tubular injury biomarkers.

Nevertheless, because the relatively mild structural injury limited our ability to evaluate a treatment-related histological effect, the present experiment does not establish structural protection by pre-ischemic SMTP-44D administration. Future studies using experimental conditions that produce reproducible and sufficiently severe structural injury, together with quantitative histopathological assessment, will be required to determine whether pre-ischemic SMTP-44D administration confers structural renal protection.

The treatment during the ischemia protocol may have limited clinical applicability compared with the treatment prior to the ischemia protocol because the onset and duration of ischemia cannot always be precisely identified. Moreover, a drug administered during renal ischemia may be distributed slowly or insufficiently to the renal tissue where it exerts its effects. Conversely, administration prior to ischemia ensures drug distribution in the tissue before the onset of the ischemic insult, thereby minimizing early reperfusion injury. Therefore, we examined the potential of pre-ischemic administration of SMTP-44D. In addition to the dose of 30 mg/kg, we evaluated a lower dose of SMTP-44D (3 mg/kg) using the treatment prior to the ischemia protocol. The partial efficacy observed at 3 mg/kg suggests pharmacological activity at the lower dose. Although the 30 mg/kg dose showed numerically greater improvements than the 3 mg/kg dose in several renal functional parameters, no statistically significant differences were observed between the two doses. Therefore, the present findings do not establish dose dependence, and neither a definitive dose–response relationship nor the optimal therapeutic dose can be determined from this study. Further studies using a wider range of doses are required to define the dose–response relationship of SMTP-44D.

There are several clinically relevant situations in which renal ischemia can be anticipated. For example, cardiac surgery involving cardiopulmonary bypass is associated with a high incidence of AKI, largely mediated by ischemia–reperfusion injury, oxidative stress, and inflammation [44,45,46,47,48]. In such contexts, prophylactic administration of renoprotective agents prior to ischemia has been proposed as a potential strategy to mitigate renal injury [49]. Therefore, the functional and molecular effects observed with pre-ischemic SMTP-44D administration provide a rationale for further investigation of SMTP-44D as a potential perioperative renoprotective strategy. However, confirmation of structural protection and optimization of the administration protocol will be required before its translational potential can be established.

The pathophysiology of I/R-induced AKI involves a complex interplay of metabolic dysfunction, oxidative stress, and inflammation. As previously described, the reperfusion phase triggers an increase in ROS levels, resulting in cellular damage and the activation of inflammatory cascades involving nuclear factor-kappa B (NF-κB) and various cytokines [50,51,52]. Consistent with this mechanism, our study revealed that I/R injury led to substantial elevations in renal TNF-α, IL-6, IL-1β, and the oxidative stress marker NOX-1 [53,54]. SMTP-44D treatment significantly suppressed TNF-α, IL-1β, and NOX-1 levels, whereas the reduction in IL-6 did not reach statistical significance, although a decreasing trend was observed and should therefore be interpreted cautiously. The mechanism underlying this protective effect is likely attributable to specific inhibition of sEH. Inhibition of sEH prevents the degradation of EETs, endogenous lipid mediators known to possess potent cytoprotective properties [55]. EETs exert anti-inflammatory effects by inhibiting NF-κB signaling and reducing oxidative stress [56,57]. Collectively, our results suggest that the functional and molecular protective effects of SMTP-44D are associated with suppression of inflammation and oxidative stress, potentially involving the sEH–EET axis.

Another key aspect of our findings relates to the changes in renal hemodynamics and tubular function, specifically UF, Ccr, and FE_Na_. In the AKI group, FE_Na_ was significantly elevated, indicating a loss of tubular reabsorptive capacity resulting from epithelial injury. SMTP-44D treatment significantly reduced FE_Na_ toward normal levels, suggesting preservation of tubular function and reabsorptive capacity. Notably, despite improved tubular reabsorption, as reflected by reduced FE_Na_, the treatment prior to the ischemia group exhibited a significant increase in UF. This increase in urine output can be explained by a restoration of glomerular filtration rate, as indicated by improved Ccr. The glomerular filtration rate depends on glomerular blood flow, which can be increased by dilating the afferent arteriole. The vasodilatory and natriuretic actions of EETs [56,58] may be responsible for this phenomenon, rather than the polyuria associated with renal failure.

The pharmacological profile of SMTP-44D offers distinct advantages over its analog, SMTP-7. In our previous study, we reported that SMTP-7 protects against AKI [27]. However, SMTP-7 possesses a dual mechanism of action, involving sEH inhibition and enhancement of plasminogen activation (thrombolytic activity) [31]. Although thrombolytic activity is beneficial for thrombotic complications, it may not be essential in perioperative settings where AKI frequently occurs. Unlike SMTP-7, SMTP-44D lacks thrombolytic activity, but retains sEH inhibitory activity. This pharmacological profile may be advantageous in settings in which thrombolytic activity is unnecessary, while retaining the potential protective effects associated with sEH inhibition. Additionally, the molecular weight of SMTP-44D (535.64) is lower than that of SMTP-7 (869.09) [25]. Although a direct pharmacokinetic comparison was not performed in this study, compounds with lower molecular weight generally exhibit improved tissue penetration and bioavailability [59].

There are certain limitations in this study. First, while our data suggest that the protective effects of SMTP-44D are associated with inflammation and oxidative stress pathways, the evidence remains associative rather than causal because we did not directly quantify EET levels in renal tissue to confirm local target engagement. Nevertheless, the observed suppression of pro-inflammatory cytokines and oxidative stress markers is consistent with the known downstream effects of sEH inhibition and EET signaling. However, based on our previous investigations of SMTP-44D, which demonstrated increased EET levels in serum but not in tissues [32], it is possible that circulating EETs contribute, at least in part, to the protective effects observed in this study. This is consistent with the known systemic actions of EETs. Second, only two dose levels were evaluated in the present study. No statistically significant differences were observed between the 3 and 30 mg/kg groups; therefore, dose dependence cannot be concluded from the present data. The effective dose range and optimal therapeutic dose of SMTP-44D for AKI remain to be established. Future studies using a wider range of doses will be required to define the dose–response relationship. Third, our observations were limited to the acute phase (24 h), and the mechanistic evaluation in this study remains limited. Although we demonstrated significant modulation of inflammatory and oxidative stress markers, these findings are associative rather than causal and do not fully elucidate the underlying molecular pathways. Future studies incorporating longer observation periods, as well as more detailed mechanistic approaches, including cell-based experiments and pathway-specific analyses, will be required to clarify the precise signaling mechanisms and long-term effects of SMTP-44D on renal repair and fibrosis. Fourth, blinding was not implemented in this study, which may introduce potential bias. However, the primary endpoints consisted of objective and quantitative measurements, which are less likely to be influenced by observer bias. Fifth, although histopathological examination supported the protective effect of SMTP-44D in the treatment during the ischemia protocol, an apparent morphological effect was not observed in the treatment prior to the ischemia protocol, in which I/R-induced structural injury was relatively mild. Therefore, structural renoprotection by pre-ischemic SMTP-44D administration cannot be concluded from the present study. Differences in fluid administration between the two experimental protocols may have contributed to the difference in overall injury severity, although the identical fluid volume and administration schedule used for vehicle- and SMTP-44D-treated animals within each protocol minimize this factor as an explanation for the treatment-related differences in functional and molecular outcomes. Because the infusion volume and administration rate differed between the two protocols, direct comparison of their relative efficacy is not appropriate. Future studies designed to compare administration timing should use matched infusion volumes and administration rates across protocols. Future studies incorporating quantitative histopathological analysis under conditions producing reproducible structural injury are required. Sixth, a selective sEH inhibitor was not included as a positive control, and a group treated with SMTP-44D alone without I/R injury was not evaluated. Therefore, the present study cannot independently determine whether SMTP-44D itself affects renal function or morphology under non-I/R conditions. In a separate 14-day repeated intravenous dosing study, no treatment-related renal toxicity was observed based on histopathological and blood biochemical assessments (un-published data). However, these findings provide only supportive safety information and do not substitute for an SMTP-44D-alone group in the present experimental setting. Accordingly, the absence of this group remains a limitation of the present study. Future studies incorporating an SMTP-44D-alone group will be required to directly assess the effects of SMTP-44D on the kidney in the absence of I/R injury and to further strengthen the safety evaluation. In addition, because currently available sEH inhibitors primarily target the C-terminal hydrolase domain, whereas SMTP-44D inhibits both the N- and C-terminal domains of sEH, an appropriate positive control with a comparable pharmacological profile has not yet been established.

Seventh, different batches of SMTP-44D were used in the two administration protocols. Although both batches were produced using the same standardized manufacturing procedure, met the same predefined quality specifications, and showed >99% purity by HPLC. However, we did not perform MS, NMR, IR and specific rotation analyses on every batch. Therefore, we cannot exclude subtle batch-dependent differences. Therefore, the two protocols should be regarded as independent experiments, and differences between them should not be interpreted as direct comparisons of treatment efficacy. In addition, SMTP-44D is not commercially available, which may limit independent replication of the present findings.

Eighth, the pharmacokinetic and cellular distribution profiles of SMTP-44D have not been characterized in this study. Although previous in vitro studies have demonstrated pharmacological effects of SMTP-44D in cultured cells [33], direct cellular uptake, intracellular concentrations, and the mechanism of membrane transport have not been determined.

In conclusion, SMTP-44D improved renal functional parameters and tubular injury biomarkers following renal I/R injury, accompanied by attenuation of inflammatory and oxidative stress responses. Structural protection was observed with treatment during ischemia, whereas pre-ischemic administration provided functional and molecular protection without demonstrable histological protection. These findings suggest that SMTP-44D may represent a potential non-thrombolytic sEH-targeting candidate for AKI. Further studies are required to establish the optimal dose and administration conditions and to determine the extent of structural protection.

## 4. Materials and Methods

### 4.1. Materials

Acetaminophen, 10% neutral buffered formalin solution (pH 7.4), and isoflurane were obtained from FUJIFILM Wako Pure Chemical Corporation (Osaka, Japan). Enrofloxacin (Baytril^®^) and a protease inhibitor cocktail were purchased from Bayer AG (Leverkusen, Germany) and Sigma-Aldrich Co., LLC (St. Louis, MO, USA), respectively. RIPA buffer was obtained from Cayman Chemical Company (Ann Arbor, MI, USA). SMTP-44D (sodium salt form; >99% purity as confirmed by HPLC), produced by *S. microspora*, was kindly provided by TMS Co., Ltd. (Tokyo, Japan). SMTP-44D used in the treatment during ischemia and treatment prior to ischemia protocols corresponded to batches 180904SON and 230508TCM, respectively. Both batches were prepared using the same standardized manufacturing procedure and met the same predefined quality specifications prior to use. The two treatment protocols were conducted as independent experiments, and no direct statistical comparison was performed between the protocols.

### 4.2. Animals

The study protocol was approved by the Institutional Animal Care and Use Committees of Showa Medical University (Approval No. 24008, 31 March 2022) and was conducted in strict accordance with the Standards Relating to the Care and Management of Experimental Animals in Japan. Male ddY mice (6–7 weeks old, 30–35 g) were obtained from Tokyo Laboratory Animals Science Co., Ltd. (Tokyo, Japan). Animals were maintained in a climate-controlled room (23 ± 2 °C, 50 ± 20% humidity) under a 12 h light–dark cycle (08:00–20:00). Food and water were provided ad libitum.

### 4.3. AKI Model Mice

The AKI model was established following previously reported methods [27]. Mice were anesthetized with isoflurane via inhalation using an anesthetic vaporizer (Nazme Co. Ltd., Tokyo, Japan) to manage to manage pain during surgery. Anesthesia was induced at 5% and maintained at 2%. Right uninephrectomy was performed via a flank incision. To ensure analgesia and prevent infection, acetaminophen (150 mg/kg, s.c.) and enrofloxacin (5 mg/kg, s.c.) were administered immediately post-surgery. Following a 2-week recovery period, the left kidney was exposed through a flank incision under isoflurane anesthesia. The renal pedicle was occluded with a nontraumatic clamp for 45 min. After ischemia, the clamp was removed to allow reperfusion. Body temperature was maintained at 37 °C using a heating lamp throughout the procedure. Mice were randomly assigned to the following groups (*n* = 8 per group): vehicle-treated (control) and SMTP-44D-treated (30 mg/kg) in the “treatment during ischemia” protocol, and vehicle-treated (control) and SMTP-44D-treated (3 or 30 mg/kg) in the “treatment prior to ischemia” protocol. The drug injection volume rate was 2 mL/kg/min (a total of 4 mL/kg for 2 min) in the treatment prior to the ischemia protocol, whereas the rate was 0.033 mL/kg for 30 min (a total of 1 mL/kg for 30 min) in the treatment during the ischemia protocol. The two administration protocols were conducted as independent experiments and were not designed for direct statistical comparison. Within each protocol, vehicle-treated and SMTP-44D-treated animals received identical infusion volumes and administration schedules. Histopathological evaluation was performed in a blinded manner. No blinding was applied during the allocation, experimental procedures, or biochemical outcome assessment. Sham-operated groups (*n* = 8) underwent identical procedures without renal clamping in both protocols.

### 4.4. Drug Administration

SMTP-44D was administered using a syringe pump (KDS200P, Muromachi Kikai, Tokyo, Japan) according to two distinct protocols. For the treatment during ischemia protocol, a total dose of SMTP-44D (30 mg/kg) was administered intravenously; an initial bolus (10% of the total dose) was injected 15 min after the onset of ischemia, followed by a continuous infusion of the remaining dose over 30 min. For the treatment prior to ischemia protocol, SMTP-44D (3 or 30 mg/kg) was infused intravenously over 2 min just before renal pedicle clamping. The maximum dose of 30 mg/kg was selected based on our previous findings, which showed dose-dependent efficacy in mouse models of diabetic nephropathy and diabetic neuropathy [32,34,35]. This dose was selected to ensure sufficient target engagement of sEH inhibition under severe I/R conditions. Additionally, a lower dose of 3 mg/kg was included in the treatment prior to the ischemia protocol to explore the effects of a lower dose. Vehicle (saline) was administered intravenously to both the sham-operated and control (I/R) groups according to the same dosing schedule as the corresponding SMTP-44D-treated groups.

### 4.5. Sample Collection

Mice were placed individually in metabolic cages 24 h after reperfusion to collect urine samples for 24 h. UF was calculated as follows: UF (μL/min/kg) = [urine volume (μL)/collection time (min)]/body weight (kg). At the study endpoint (following the 24 h urine collection), mice were euthanized by exsanguination via the abdominal aorta under deep isoflurane anesthesia (5% via inhalation using a vaporizer). Humane endpoints were predefined as rapid and irreversible weight loss (>20% within a few days) requiring euthanasia via CO_2_ inhalation; however, no animals met these criteria during the observation period. Serum was separated via centrifugation at 800× g for 15 min at 25 °C and stored at −20 °C until analysis. The left kidney was harvested and weighed to determine the KW relative to body weight (mg/g). The kidney tissue was preserved for analysis of inflammatory and oxidative stress markers. Kidney tissues obtained from the same animals used for the urinary and serum biochemical analyses in both experimental protocols were also subjected to histopathological evaluation.

### 4.6. Histological Study

Kidney tissues fixed in 10% neutral-buffered formalin were embedded in paraffin, cut into 3 μm sections, and stained with H.E. Histopathological examination was conducted using an optical microscope (BHS, Olympus Corp., Tokyo, Japan) equipped with a digital camera (Olympus digital camera E-330, Olympus Corp., Tokyo, Japan). Renal tubular injury was histopathologically evaluated in the outer stripe of the outer medulla, where I/R-induced injury was prominently observed. Morphological alterations, including tubular dilation, tubular necrosis, tubular cast formation, and inflammatory cell infiltration, were assessed according to the histopathological criteria described by Solez et al. [60]. Representative H.E.-stained images were selected to illustrate the morphological features observed in each experimental group. Histopathological evaluation was performed in a blinded manner.

### 4.7. Parameters of Renal Function

Ualb levels were quantified using an Albuwell M ELISA Kit (Ethos Biosciences, Inc., Logan Township, PA, USA) as previously described [27,36] and in accordance with the manufacturer’s protocol. Urine samples were diluted 30-fold with the supplied normal horse serum containing bovine serum albumin diluent. Albumin concentration was determined from a standard curve, and Ualb was calculated as follows: Ulab (mg/h/kg) = [concentration of albumin in urine (μg/mL) × urine volume (μL)]/[urine collection time (h) × mouse body weight (kg) × 10^6^]. BUN was assessed using a QuantiChrom^TM^ Urea Assay Kit (BioAssay Systems, Hayward, CA, USA) as described previously [27] and following the manufacturer’s protocol. Serum samples were diluted with distilled water according to the expected concentration range: 3-fold for the sham-operated, 10-fold for the vehicle-treated AKI, and 5-fold for the drug-treated AKI groups. BUN concentrations (mg/dL) were determined from a standard curve. Scr and creatinine concentrations in urine (Ucr) were measured using a LabAssay^TM^ Creatinine Kit (FUJIFILM Wako Pure Chemical Corporation, Osaka, Japan), following the manufacturer’s instructions and a previously reported protocol [27]. Urine samples were diluted 10-fold with distilled water, while serum samples were analyzed without dilution. Scr (mg/dL) and Ucr (mg/dL) concentrations were determined from a standard curve. Ccr was calculated as follows: Ccr (mL/min/kg) = [Ucr (mg/dL) × UF (μL/min/kg)]/[Scr (mg/dL) × 1000]. Sodium concentrations in serum (S_Na_) and urine (U_Na_) were analyzed using a Fuji DRI-CHEM 800 analyzer (FUJIFILM Corporation, Tokyo, Japan) as previously described [27]. Urine samples were diluted 2-fold with distilled water, and serum samples were used undiluted. FE_Na_ was calculated as follows: FE_Na_ (%) = [U_Na_ (mEq/L) × UF (μL/min/kg)]/[S_Na_ (mEq/L) × Ccr (mL/min/kg) × 1000] × 100.

### 4.8. Assessment of Tubular Damage

To evaluate renal tubular injury, urinary levels of NGAL and KIM-1 were measured using Quantikine ELISA kits (R&D Systems Inc., Minneapolis, MN, USA) following the manufacturer’s instructions and a previously described protocol [27]. Urine samples were diluted 30-fold with distilled water. Concentrations were determined from standard curves and normalized to Ucr levels. Data are expressed as ng/μg or pg/μg creatinine in urine, as previously described [27,61,62].

### 4.9. Assessment of Renal Inflammatory and Oxidative Stress Markers

Frozen kidney tissues were homogenized in 500 μL of RIPA buffer containing a protease inhibitor cocktail. The homogenates were centrifuged at 10,000× g for 15 min at 4 °C, and the supernatants were collected for analysis. Levels of inflammatory signaling markers, including TNF-α, IL-6, and IL-1β in kidney tissue, were measured using Quantikine ELISA kits (R&D Systems Inc., Minneapolis, MN, USA). Levels of the oxidative stress marker NOX-1 were measured using NOX-1 ELISA kits (MyBioSource Inc., San Diego, CA, USA) following the manufacturer’s instructions and a previously described protocol [27]. Prior to the assays, supernatants were diluted with RIPA buffer containing protease inhibitors as follows: 2-fold for TNF-α, 3-fold for IL-1β, 5-fold for NOX-1, and 20-fold for total protein measurement, whereas IL-6 was measured in undiluted samples. Total protein concentrations were determined using the Pierce™ bicinchoninic acid protein assay kit (Thermo Fisher Scientific Inc., Minneapolis, MN, USA) with 20-fold diluted samples, following the manufacturer’s instructions and a previously outlined protocol [27,36]. Concentrations were determined from standard curves, normalized to total protein content, and expressed as pg/mg protein.

### 4.10. Statistical Analysis

Data are expressed as the mean ± standard error of the mean. Statistical differences between groups were determined using one-way analysis of variance followed by the Bonferroni post hoc test. A *p*-value of <0.05 was considered statistically significant.

## Figures and Tables

**Figure 1:**
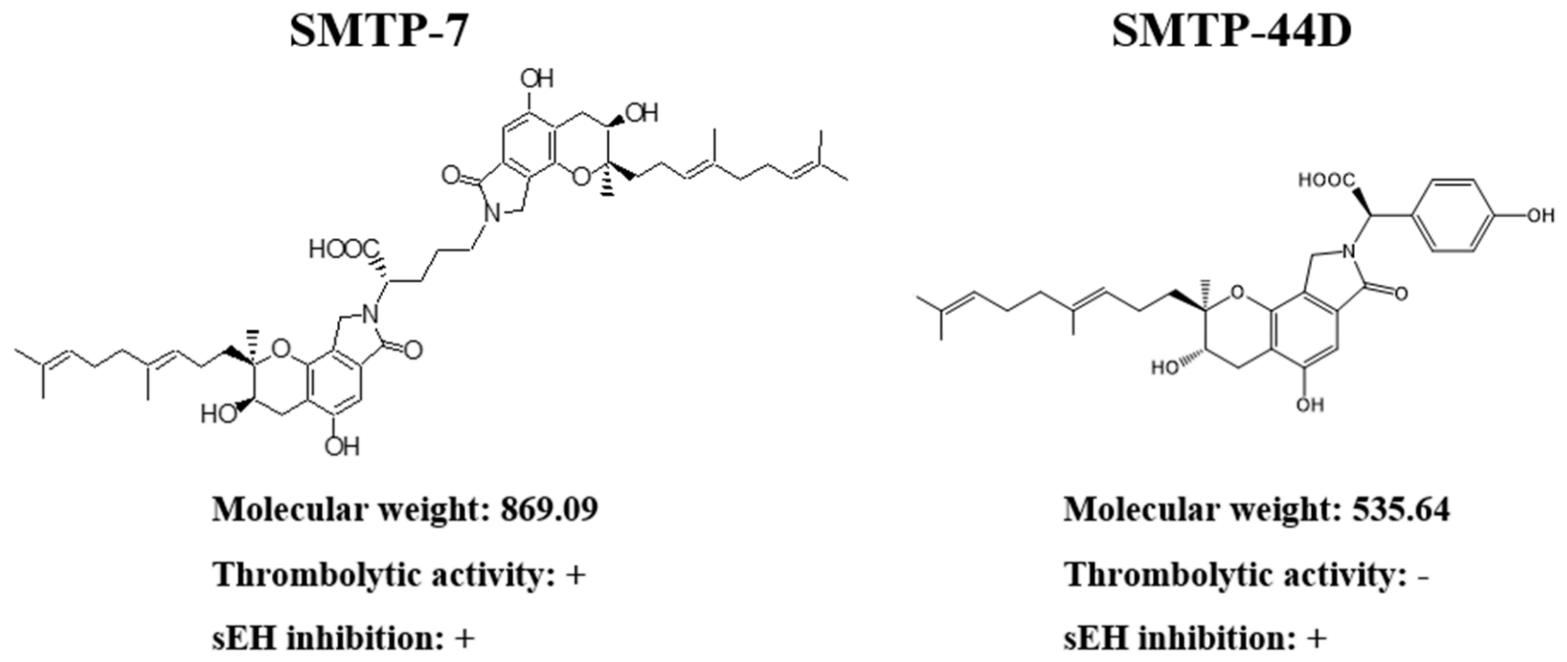
Chemical structures of SMTP-7 and SMTP-44D.

**Figure 2:**
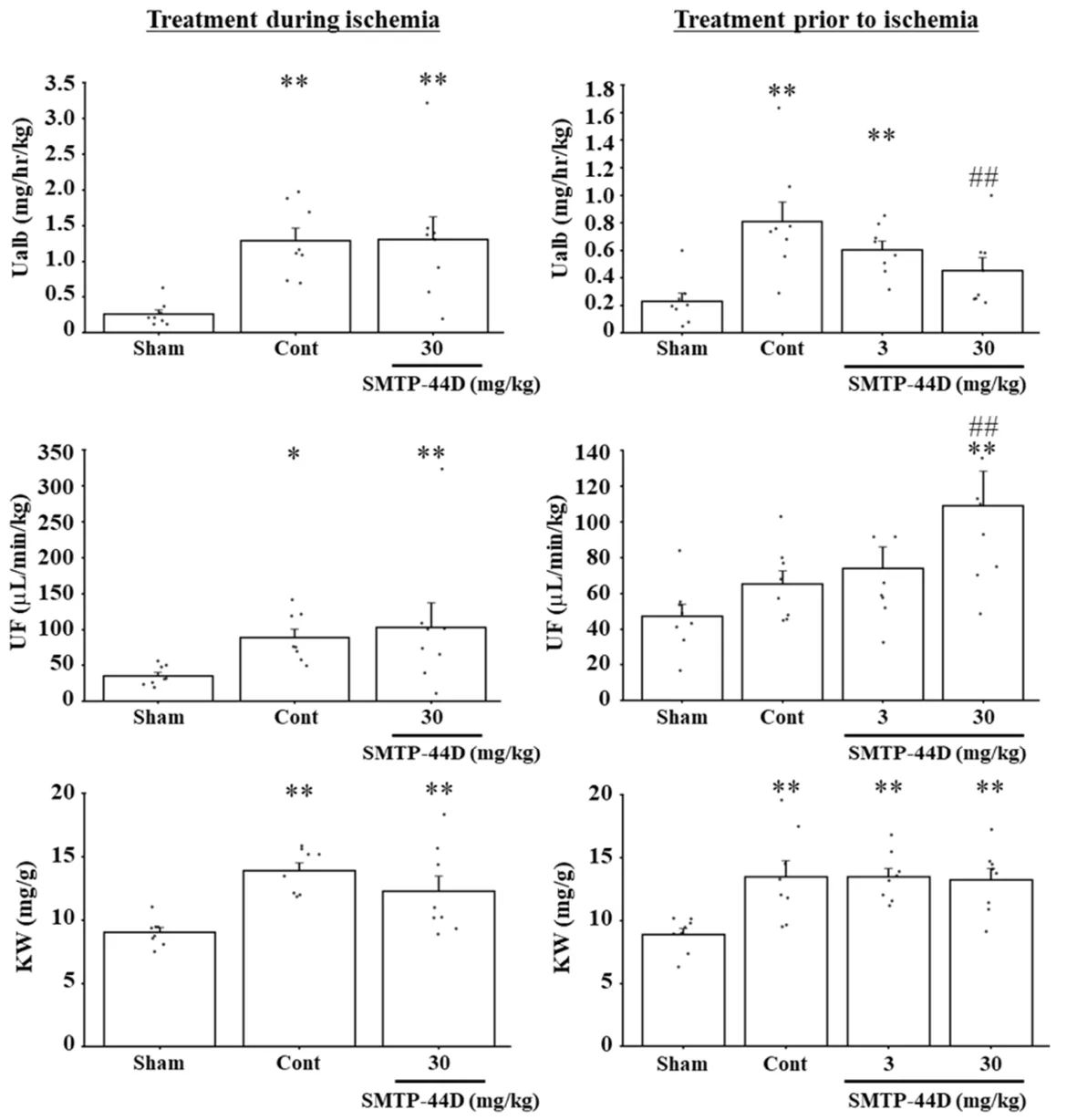
Effects of SMTP-44D on Ualb, UF, and KW in AKI model mice. SMTP-44D was administered at a dose of 30 mg/kg during ischemia or at either 3 mg/kg or 30 mg/kg prior to ischemia. Data are presented as the mean ± SEM from eight mice, and individual data points are shown for each group. * *p* < 0.05 and ** *p* < 0.01 vs. the sham-operated group. ^##^ *p* < 0.01 vs. the control group.

**Figure 3:**
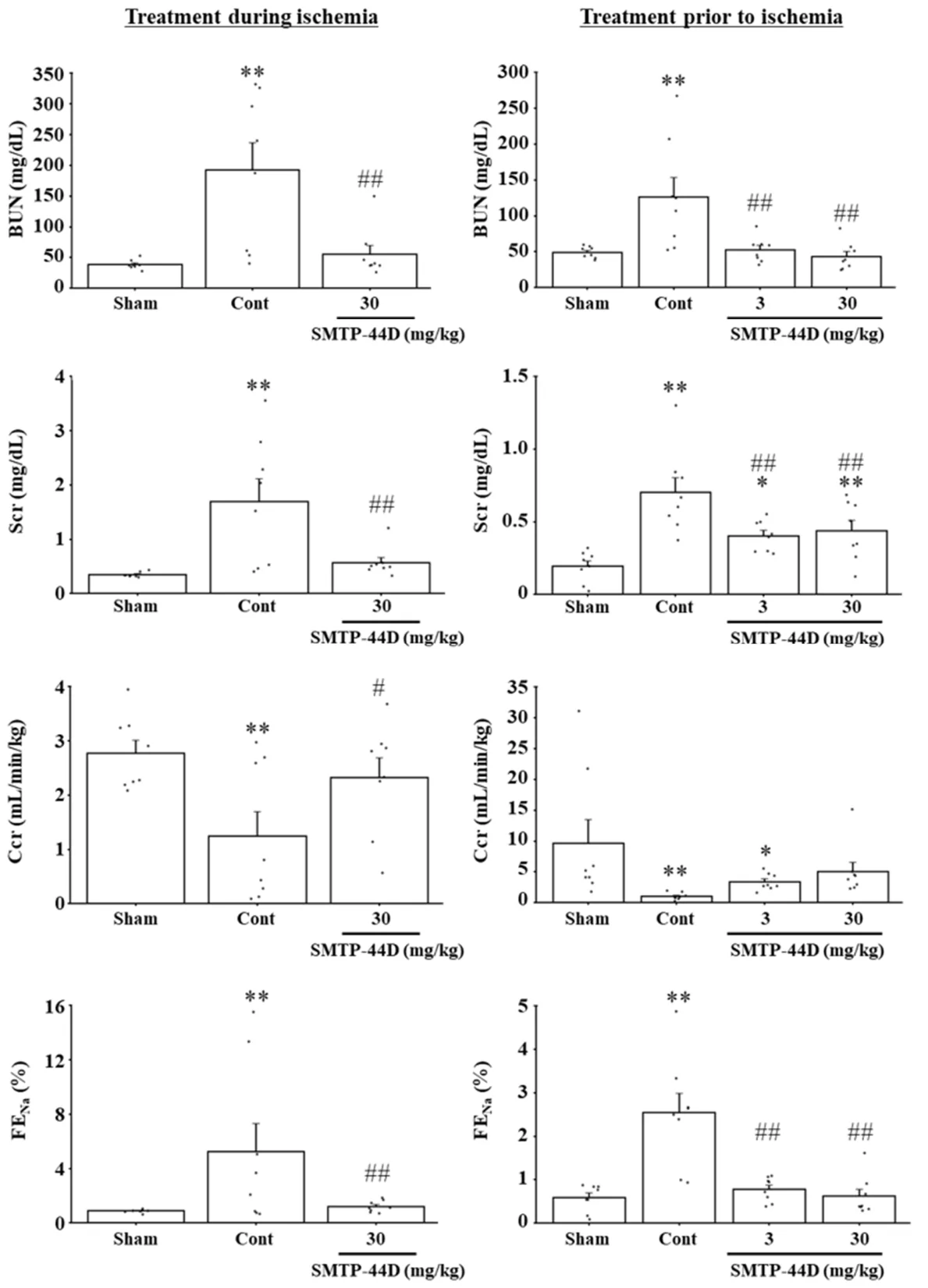
Effects of SMTP-44D on Scr, Ccr, and FE_Na_ in AKI model mice. SMTP-44D was administered at a dose of 30 mg/kg during ischemia or at either 3 mg/kg or 30 mg/kg prior to ischemia. Data are presented as the mean ± SEM from eight mice, and individual data points are shown for each group. * *p* < 0.05 and ** *p* < 0.01 vs. the sham-operated group. ^#^ *p* < 0.05 and ^##^ *p* < 0.01 vs. the control group.

**Figure 4:**
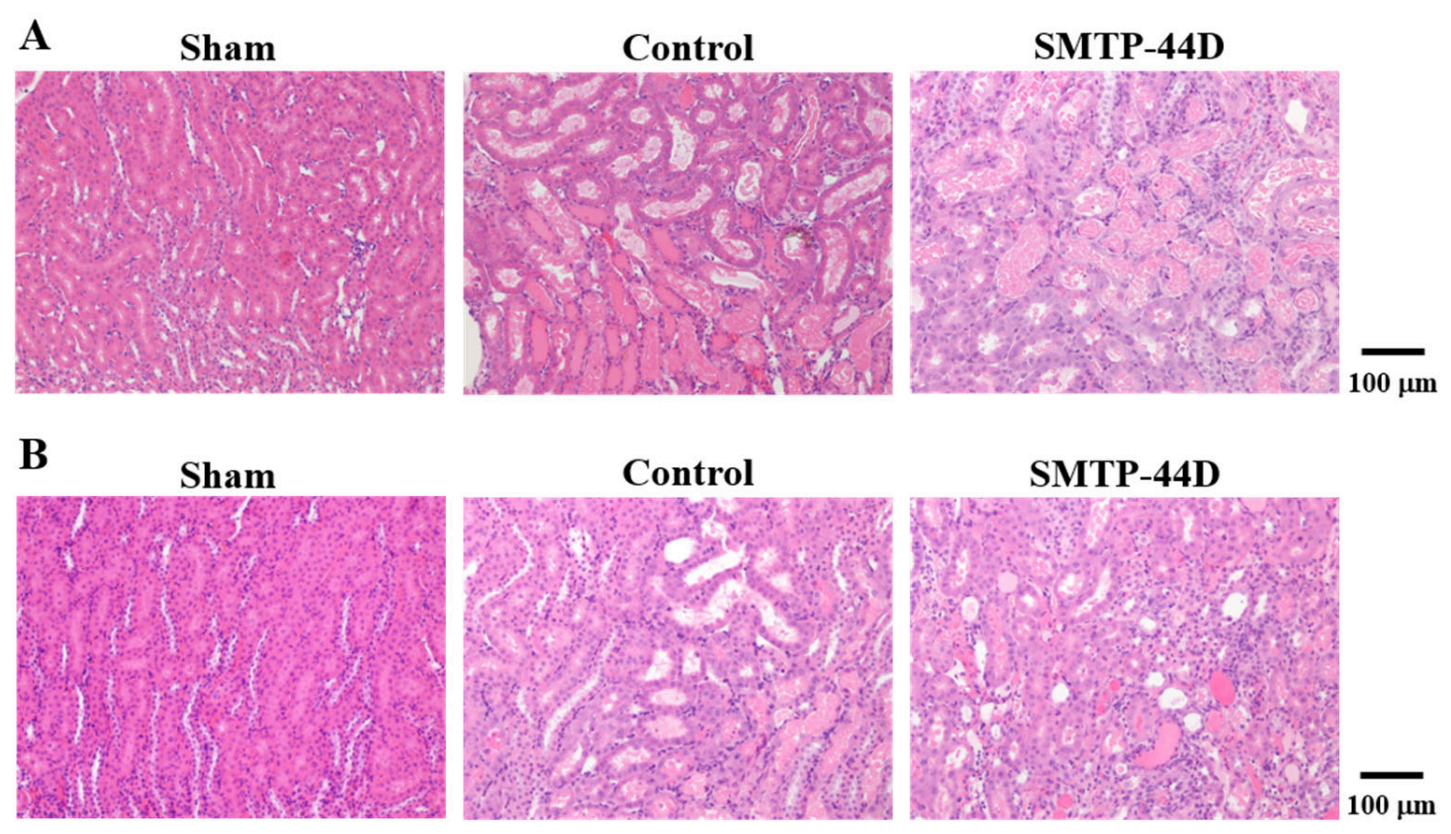
Effects of SMTP-44D on renal histopathological injury following ischemia–reperfusion. Representative H.E.-stained kidney sections of the outer stripe of the outer medulla in the treatment during ischemia protocol (**A**) and the treatment prior to ischemia protocol (**B**). Kidney tissues were obtained from the same animals used for the urinary and serum biochemical analyses in the present study. Scale bar = 100 μm.

**Figure 5:**
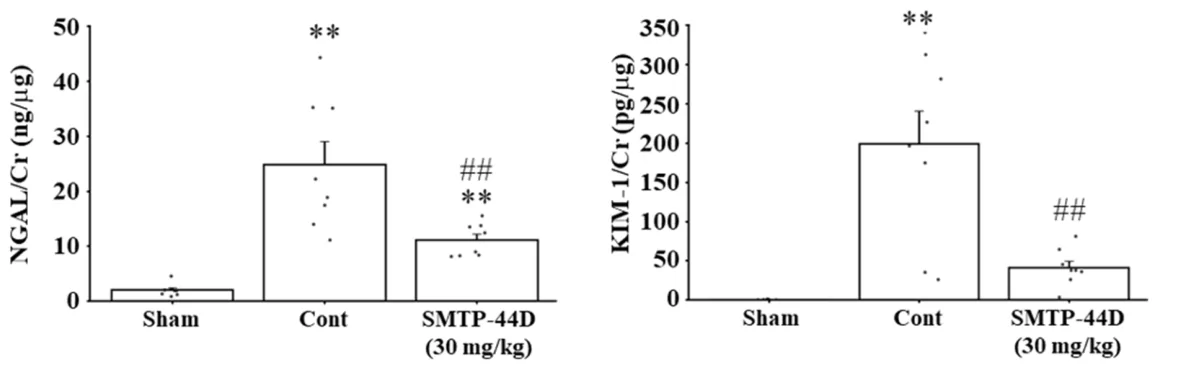
Effects of SMTP-44D on urinary levels of NGAL and KIM-1 in AKI model mice. SMTP-44D (30 mg/kg) was administered prior to ischemia. Data are presented as the mean ± SEM from eight mice, and individual data points are shown for each group. ** *p* < 0.01 vs. the sham-operated group. ^##^ *p* < 0.01 vs. the control group.

**Figure 6:**
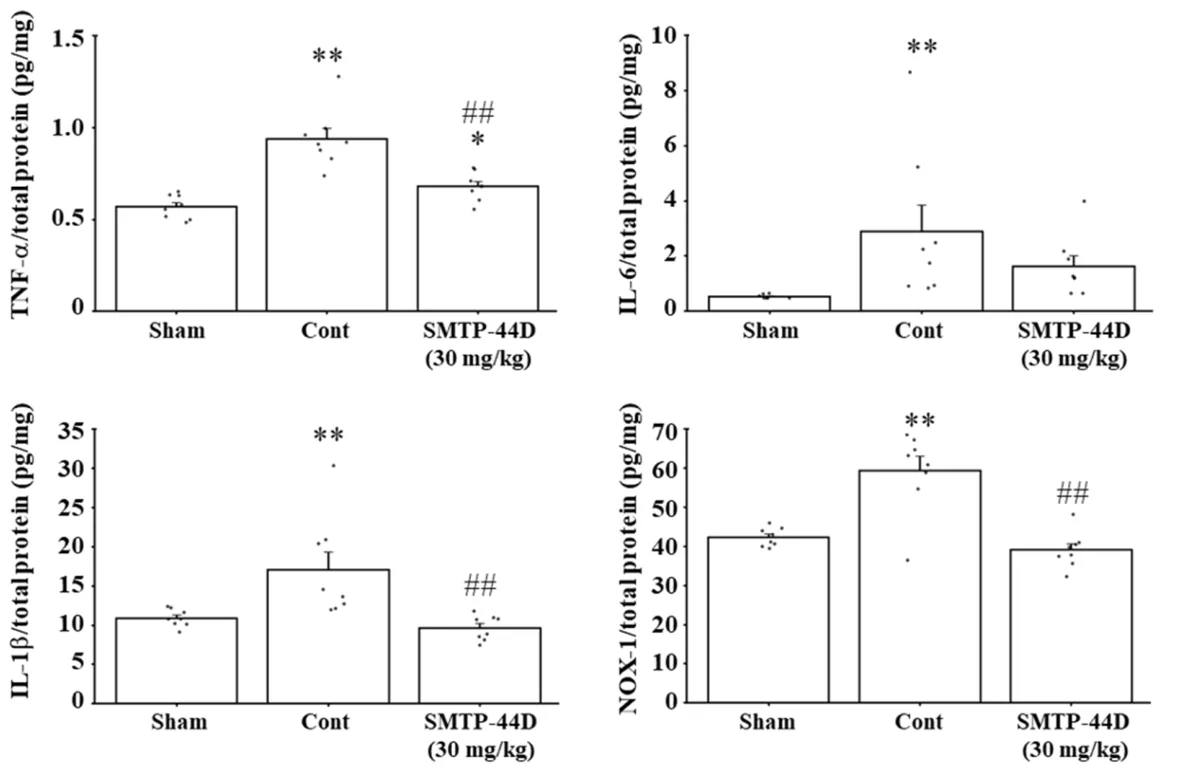
Effects of SMTP-44D on the levels of TNF-α, IL-6, IL-1β, and NOX-1 in kidney tissues from AKI model mice. SMTP-44D (30 mg/kg) was administered prior to ischemia. Data are presented as the mean ± SEM from eight mice, and individual data points are shown for each group. * *p* < 0.05 and ** *p* < 0.01 vs. the sham-operated group. ^##^ *p* < 0.01 vs. the control group.

## Data Availability

The data that support the findings of this study are available from the corresponding author upon reasonable request.

## Abbreviations

The following abbreviations are used in this manuscript:

AKI	acute kidney injury
I/R	ischemia–reperfusion
SMTP-44D	*Stachybotrys microspora* triprenyl phenol-44D
ROS	reactive oxygen species
SMTPs	*Stachybotrys microspora* triprenyl phenols
sEH	soluble epoxide hydrolase
EETs	epoxyeicosatrienoic acids
Ualb	urinary albumin
UF	Urine flow
KW	kidney weight
BUN	blood urea nitrogen
Scr	creatinine concentrations in serum
FE_Na_	fractional excretion of sodium
Ccr	creatinine clearance
H.E.	hematoxylin and eosin
NGAL	neutrophil gelatinase-associated lipocalin
KIM-1	kidney injury molecule 1
TNF-α	tumor necrosis factor alpha
IL	interleukin
NOX-1	NADPH oxidase-1
Ucr	creatinine concentrations in urine

## References

[1] Brown J.R., Hisey W.M., Marshall E.J., Likosky D.S., Nichols E.L., Everett A.D., Pasquali S.K., Jacobs M.L., Jacobs J.P., Parikh C.R. (2016). Acute Kidney Injury Severity and Long-Term Readmission and Mortality After Cardiac Surgery. Ann. Thorac. Surg..

[2] Schulman I.H., Chan K., Der J.S., Wilkins K.J., Corns H.L., Sayer B., Ngo D.A., Eggers P., Norton J., Shah N. (2023). Readmission and Mortality After Hospitalization With Acute Kidney Injury. Am. J. Kidney Dis..

[3] Meng X.M., Wang L., Nikolic-Paterson D.J., Lan H.Y. (2025). Innate immune cells in acute and chronic kidney disease. Nat. Rev. Nephrol..

[4] Hoste E.A., Bagshaw S.M., Bellomo R., Cely C.M., Colman R., Cruz D.N., Edipidis K., Forni L.G., Gomersall C.D., Govil D. (2015). Epidemiology of acute kidney injury in critically ill patients: The multinational AKI-EPI study. Intensive Care Med..

[5] Kidney Disease: Improving Global Outcomes (KDIGO) Acute Kidney Injury Work Group (2012). KDIGO Clinical Practice Guideline for Acute Kidney Injury. Kidney Int. Suppl..

[6] Gonsalez S.R., Cortês A.L., Silva R.C.D., Lowe J., Prieto M.C., Silva Lara L.D. (2019). Acute kidney injury overview: From basic findings to new prevention and therapy strategies. Pharmacol. Ther..

[7] Basile D.P., Anderson M.D., Sutton T.A. (2012). Pathophysiology of acute kidney injury. Compr. Physiol..

[8] Kellum J.A., Prowle J.R. (2018). Paradigms of acute kidney injury in the intensive care setting. Nat. Rev. Nephrol..

[9] Mathis M.R., Mentz G.B., Cao J., Balczewski E.A., Janda A.M., Likosky D.S., Schonberger R.B., Hawkins R.B., Heung M., Ailawadi G. (2025). Hospital and clinician practice variation in cardiac surgery and postoperative acute kidney injury. JAMA Netw. Open.

[10] Nadim M.K., Forni L.G., Bihorac A., Hobson C., Koyner J.L., Shaw A., Arnaoutakis G.J., Ding X., Engelman D.T., Gasparovic H. (2018). Cardiac and Vascular Surgery-Associated Acute Kidney Injury: The 20th International Consensus Conference of the ADQI (Acute Disease Quality Initiative) Group. J. Am. Heart Assoc..

[11] Horton R., Berman P. (2015). Eliminating acute kidney injury by 2025: An achievable goal. Lancet.

[12] Panisello-Roselló A., Roselló-Catafau J., Adam R. (2020). New insights in molecular mechanisms and pathophysiology of ischemia-reperfusion injury 2.0: An updated overview. Int. J. Mol. Sci..

[13] Linkermann A., Chen G., Dong G., Kunzendorf U., Krautwald S., Dong Z. (2014). Regulated cell death in AKI. J. Am. Soc. Nephrol..

[14] Yao C., Li Z., Sun K., Zhang Y., Shou S., Jin H. (2024). Mitochondrial dysfunction in acute kidney injury. Ren. Fail..

[15] Nath K.A., Norby S.M. (2000). Reactive oxygen species and acute renal failure. Am. J. Med..

[16] Lu Q., Wang M., Gui Y., Hou Q., Gu M., Liang Y., Xiao B., Zhao A.Z., Dai C. (2020). Rheb1 protects against cisplatin-induced tubular cell death and acute kidney injury via maintaining mitochondrial homeostasis. Cell Death Dis..

[17] Zhaoo Z., Wu J., Xu H., Zhou C., Han B., Zhu H., Hu Z., Ma Z., Ming Z., Yao Y. (2020). XJB-5-131 inhibited ferroptosis in tubular epithelial cells after ischemia-reperfusion injury. Cell Death Dis..

[18] Mourad B.H., Ashour Y.A. (2020). Demonstration of Subclinical Early Nephrotoxicity Induced by Occupational Exposure to Silica among Workers in Pottery Industry. Int. J. Occup. Environ. Med..

[19] Ronco C., Bellomo R., Kellum J.A. (2019). Acute kidney injury. Lancet.

[20] Tian Z., Ni R., Zheleznova N.N., Allen-Gipson D., Wang L., Subramanian V., Dhanireddy K., Yuan S.Y., Soto N.H., Herazo-Maya J.D. (2025). Acute kidney injury induces lung damage via mitochondrial DAMPs by activating TREM-1 and cGAS-STING pathways. Cells.

[21] Kellum J.A., Romagnani P., Ashuntantang G., Ronco C., Zarbock A., Anders H.J. (2021). Acute kidney injury. Nat. Rev. Dis. Primers.

[22] Ojo B., Campbell C.H. (2022). Perioperative acute kidney injury: Impact and recent update. Curr. Opin. Anaesthesiol..

[23] Zhu X.X., Meng X.Y., Zhang A.Y., Zhao C.Y., Wei Y.T., Wen Y.Y., Su J.B., Fu X., Chen G., Xu A.J. (2025). Inhibition of PGK1 ameliorates acute kidney injury through inactivating the PKM2/ALOX12/ferroptosis pathway in a study with male mice. Nat. Commun..

[24] Hasumi K., Yamamichi S., Harada T. (2010). Small-molecule modulators of zymogen activation in the fibrinolytic and coagulation systems. FEBS J..

[25] Shibata K., Hashimoto T., Hasumi K., Honda K., Nobe K. (2018). Evaluation of the effects of a new series of SMTPs in the acetic acid-induced embolic cerebral infarct mouse model. Eur. J. Pharmacol..

[26] Akamatsu Y., Saito A., Fujimura M., Shimizu H., Mekawy M., Hasumi K., Tominaga T. (2011). *Stachybotrys microspora* triprenyl phenol-7, a novel fibrinolytic agent, suppresses superoxide production, matrix metalloproteinase-9 expression, and thereby attenuates ischemia/reperfusion injury in rat brain. Neurosci. Lett..

[27] Shibata K., Hashimoto T., Hasumi K., Nobe K. (2021). Potent efficacy of *Stachybotrys microspora* triprenyl phenol-7, a small molecule having anti-inflammatory and antioxidant activities, in a mouse model of acute kidney injury. Eur. J. Pharmacol..

[28] Morisseau C., Hammock B.D. (2013). Impact of soluble epoxide hydrolase and epoxyeicosanoids on human health. Annu. Rev. Pharmacol. Toxicol..

[29] Hasumi K., Suzuki E. (2021). Impact of SMTP Targeting Plasminogen and Soluble Epoxide Hydrolase on Thrombolysis, Inflammation, and Ischemic Stroke. Int. J. Mol. Sci..

[30] Matsumoto N., Suzuki E., Ishikawa M., Shirafuji T., Hasumi K. (2014). Soluble epoxide hydrolase as an anti-inflammatory target of the thrombolytic stroke drug SMTP-7. J. Biol. Chem..

[31] Niizuma K., Nishimura N., Hasegawa K., Moritoyo T., Kudo K., Bell J., Wald M., Umeda Y., Kuribayashi K., Toda Y. (2024). Anti-inflammatory thrombolytic JX10 (TMS-007) in late presentation of acute ischemic stroke. Stroke.

[32] Aoki A., Shibata K., Shinouchi R., Hasumi K., Nobe K. (2025). SMTP-44D prevents negative symptoms of diabetic neuropathy by inhibiting sciatic nerve apoptosis. J. Pharmacol. Sci..

[33] Shinouchi R., Shibata K., Jono S., Hasumi K., Nobe K. (2022). SMTP-44D Exerts Antioxidant and Anti-Inflammatory Effects through Its Soluble Epoxide Hydrolase Inhibitory Action in Immortalized Mouse Schwann Cells upon High Glucose Treatment. Int. J. Mol. Sci..

[34] Shinouchi R., Shibata K., Hashimoto T., Jono S., Hasumi K., Nobe K. (2020). SMTP-44D improves diabetic neuropathy symptoms in mice through its antioxidant and anti-inflammatory activities. Pharmacol. Res. Perspect..

[35] Ishibashi M., Shibata K., Terasaki M., Saito Y., Yamagishi S.I., Hasumi K., Nobe K. (2025). SMTP-44D alleviates diabetic retinopathy by suppressing inflammation and oxidative stress in in vivo and in vitro models. J. Pharmacol. Sci..

[36] Awane T., Sasaki H., Shibata K., Hasumi K., Nobe K. (2026). SMTP-44D Exerts Renoprotective Effects against Diabetic Nephropathy via Its Anti-inflammatory and Antioxidant Action. Biol. Pharm. Bull..

[37] Mishra J., Dent C., Tarabishi R., Mitsnefes M.M., Ma Q., Kelly C., Ruff S.M., Zahedi K., Shao M., Bean J. (2005). Neutrophil gelatinase-associated lipocalin (NGAL) as a biomarker for acute renal injury after cardiac surgery. Lancet.

[38] Vaidya V.S., Ferguson M.A., Bonventre J.V. (2008). Biomarkers of acute kidney injury. Annu. Rev. Pharmacol. Toxicol..

[39] Han W.K., Bailly V., Abichandani R., Thadhani R., Bonventre J.V. (2002). Kidney Injury Molecule-1 (KIM-1): A novel biomarker for human renal proximal tubule injury. Kidney Int..

[40] Ronco C., Kellum J.A., Haase M. (2012). Subclinical AKI is still AKI. Crit. Care.

[41] Wen Y., Parikh C.R. (2021). Current concepts and advances in biomarkers of acute kidney injury. Crit. Rev. Clin. Lab. Sci..

[42] Collett J.A., Corridon P.R., Mehrotra P., Kolb A.L., Rhodes G.J., Miller C.A., Molitoris B.A., Pennington J.G., Sandoval R.M., Atkinson S.J. (2017). Hydrodynamic Isotonic Fluid Delivery Ameliorates Moderate-to-Severe Ischemia-Reperfusion Injury in Rat Kidneys. J. Am. Soc. Nephrol..

[43] Prowle J.R., Kirwan C.J., Bellomo R. (2014). Fluid management for the prevention and attenuation of acute kidney injury. Nat. Rev. Nephrol..

[44] Thakar C.V., Worley S., Arrigain S., Yared J.P., Paganini E.P. (2005). Influence of renal dysfunction on mortality after cardiac surgery: Modifying effect of preoperative renal function. Kidney Int..

[45] Rosner M.H., Okusa M.D. (2006). Acute kidney injury associated with cardiac surgery. Clin. J. Am. Soc. Nephrol..

[46] Zuk A., Bonventre J.V. (2016). Acute Kidney Injury. Annu. Rev. Med..

[47] Hobson C.E., Yavas S., Segal M.S., Schold J.D., Tribble C.G., Layon A.J., Bihorac A. (2009). Acute kidney injury is associated with increased long-term mortality after cardiothoracic surgery. Circulation.

[48] Ortega-Loubon C., Tamayo E., Jorge-Monjas P. (2022). Cardiac Surgery-Associated Acute Kidney Injury: Current Updates and Perspectives. J. Clin. Med..

[49] Joannidis M., Druml W., Forni L.G., Groeneveld A.B.J., Honore P.M., Hoste E., Ostermann M., Oudemans-van Straaten H.M., Schetz M. (2017). Prevention of acute kidney injury and protection of renal function in the intensive care unit: Update 2017: Expert opinion of the Working Group on Prevention, AKI section, European Society of Intensive Care Medicine. Intensive Care Med..

[50] Fuquay R., Renner B., Kulik L., McCullough J.W., Amura C., Strassheim D., Pelanda R., Torres R., Thurman J.M. (2013). Renal ischemia-reperfusion injury amplifies the humoral immune response. J. Am. Soc. Nephrol..

[51] Xu N., Jiang S., Persson P.B., Persson E.A.G., Lai E.Y., Patzak A. (2020). Reactive oxygen species in renal vascular function. Acta. Physiol..

[52] Reid S., Scholey J.W. (2021). Recent Approaches to Targeting Canonical NF κ B Signaling in the Early Inflammatory Response to Renal IRI. J. Am. Soc. Nephrol..

[53] Akcay A., Nguyen Q., Edelstein C. (2009). Mediators of inflammation in acute kidney injury. Mediat. Inflamm..

[54] Gill P.S., Wilcox C.S. (2006). NADPH oxidases in the kidney. Antioxid. Redox Signal..

[55] Gao P., Cao Y., Ma L. (2024). Regulation of soluble epoxide hydrolase in renal-associated diseases: Insights from potential mechanisms to clinical researches. Front. Endocrinol..

[56] Node K., Huo Y., Ruan X., Yang B., Spiecker M., Ley K., Zeldin D.C., Liao J.K. (1999). Anti-inflammatory properties of cytochrome P450 epoxygenase-derived eicosanoids. Science.

[57] Liu J.Y. (2019). Inhibition of Soluble Epoxide Hydrolase for Renal Health. Front. Pharmacol..

[58] Imig J.D., Zhao X., Capdevila J.H., Morisseau C., Hammock B.D. (2002). Soluble epoxide hydrolase inhibition lowers arterial blood pressure in angiotensin II hypertension. Hypertension.

[59] Zhu R., Chen B., Bai Y., Miao T., Rui L., Zhang H., Xia B., Li Y., Gao S., Wang X.D. (2020). Lycopene in protection against obesity and diabetes: A mechanistic review. Pharmacol. Res..

[60] Solez K., Kramer E.C., Fox J.A., Heptinstall R.H. (1974). Medullary plasma flow and intravascular leukocyte accumulation in acute renal failure. Int. J. Kidney Int..

[61] Chiazza F., Chegaev K., Rogazzo M., Cutrin J.C., Benetti E., Lazzarato L., Fruttero R., Collino M. (2015). A nitric oxide-donor furoxan moiety improves the efficacy of edaravone against early renal dysfunction and injury evoked by ischemia/reperfusion. Oxid. Med. Cell Longev..

[62] Nørgård M.Ø., Svenningsen P. (2023). Acute Kidney Injury by Ischemia/Reperfusion and Extracellular Vesicles. Int. J. Mol. Sci..

